# The effect of refractive index of fillers and polymer matrix on translucency and color matching of dental resin composite

**DOI:** 10.1080/26415275.2021.1906879

**Published:** 2021-04-01

**Authors:** Maija Oivanen, Filip Keulemans, Sufyan Garoushi, Pekka K. Vallittu, Lippo Lassila

**Affiliations:** a Department of Biomaterials Science and Turku Clinical Biomaterials Center -TCBC, Institute of Dentistry, University of Turku, Turku, Finland; b City of Turku Welfare Division, Oral Health Care, Turku, Finland

**Keywords:** Refraction index, translucency, chameleon effect, color matching

## Abstract

**Objective:**

When restorative resin composites absorb light from the surrounding tooth structures, it creates a color-match, which is known as ‘a chameleon effect’. In this study, series of co-monomer mixtures were prepared with an increasing refraction index (RI) and mixed with glass fillers. The aim of this study was to optimize the mismatch of RI of resin/fillers to create the chameleon effect.

**Materials and Methods:**

BisGMA/TEGDMA resins were prepared with seven different mix fractions from 20 to 80%. Two different series (A&B) of submicron (Ø 0.7 μm) silanized fillers (70 wt%) (A: Schott RI = 1.53, B: Esschem RI = 1.54) were mixed with resins (30 wt%). Disc-shaped specimens (1 mm thickness, Ø10 mm) for each composite combination (*n* = 3) were prepared and light cured for 20 s. Commercial resin composite (OmniChroma, Tokuyama Dental) was used as control. The translucency parameter (TP) was measured using a spectrophotometer. The color matching abilities of the experimental composites were visually analyzed. Data were statistically analyzed using ANOVA.

**Results:**

The composition of resin and type of fillers had a statistically significant effect on TP values (*p* < .05). The highest TP values were achieved around 50%-50% fractions of Bis-GMA and TEGDMA for series A and around 60%-40% fraction of Bis-GMA and TEGDMA for series B. Data showed that a high or low fraction of BisGMA resulted in a low translucent composite. Experimental resin composite (80% Bis-GMA) from series A was behaving similarly to Omnichroma in reference to TP values and color matching.

**Conclusions:**

Including fillers with RI of 1.53 into BisGMA/TEGDMA resin with RI of 1.524 resulted in composite resin providing a good color match with surrounding structure ‘chameleon effect’.

## Introduction

One of the key objectives of esthetic restorative dentistry is to create restorations that complement the natural tooth's optical properties. Color, translucency, opalescence and fluorescence are optical properties that give the natural tooth its essential appearance [1]. Translucency and color have the highest effect on the natural tooth appearance among these esthetic characteristics, since they are the most easily observed [2]. The color difference of natural teeth promoted manufacturers to develop resin composite systems that involve many colors where layering techniques have been suggested [3]. In addition, resin composites are accessible in multiple opacities, typically referred to as dentin, body or opaque and enamel or translucent, with the goal of mimicking the dentin and enamel optical properties [3].

It was stated that regular dental practitioners working in public dental services consume over half of their working hours applying direct resin composite restorations [4]. Accordingly, dental practitioners are always searching for an effective method to handle their clinical procedures involving simple techniques and reduced practical steps to decrease total time and costs. This trend or wish of shortening the restorative procedure time and simplifying color matching leads to competition between dental manufacturers to develop a universal resin composite (single shade), which could possibly match a wide range of classical shades [5].

It has been stated that the materials showing sufficient light diffusivity, i.e. translucency within the resin composite are capable to produce a chameleon effect attributed to the reflection of the adjacent tooth color and transference of its own color into the nearby tooth structure, which would lead to enhanced color matching [6,7]. However, the chameleon effect of resin composites was influenced also by the variation in the initial color and size of the restoration [8].

Translucency could be defined as middle state of opacity and transparency [9]. A translucent material permits light to pass into its structure, but it disperses light in comparison with transparent material, preventing the objects behind it from being clearly observed [9,10]. A shift in the refractive index usually results in a shift in the direction of light [11]. In case of materials containing different components, such as resin composites, the different components like the inorganic fillers and the resin matrix are needed to have similar refractive indices (RI) in order to become highly translucent [11]. The effect of the loading ratio, type and particle size of the fillers on the appearance of resin composites has been explored in literature in several studies [12,13]. Resin composites additionally include an organic matrix with a RI that varies from those of the inorganic fillers. The refractive index of the fillers should vary from 1.47 to 1.52 and match that of the cured resin matrix [13]. If there is a mismatch between the RI of the filler and the resin matrix, the filler may rise the opacity of the materials owing to extreme refraction and reflection at the filler-matrix interfaces [11]. This research studied the influence of RI mismatch of resin/fillers components on translucency and chameleon effect (color matching) of experimental resin composite.

The null hypothesis was that there is no difference in the translucency and color matching between experimental resin composites regardless of RI mismatch of resin/fillers components.

## Materials and methods

### Materials

Through Esstech Inc. (Essington, PA, USA) TEGDMA (RI = 1.46) and Bis-GMA (RI = 1.54) were acquired. From Sigma-Aldrich (St. Louis, MO, USA) N,N’-dimethylaminoethyl methacrylate (DMAEMA), and camphoroquinone (CQ) were gained. The used reagents were not specifically purified. Two types of silanated Silica fillers BaAlSiO_2_ (Ø 0.7 µm) with different RIs (1.53 and 1.54) were obtained from Schott (UltraFine, Schott, Landshut, Germany) and Esschem (Esschem Europe LTD, Seaham, England) respectively. Commercial single-shade (utilizes chameleon effect) dental resin composite (OmniChroma, Tokuyama Dental Corp., Japan) was used as control.

### Preparation of the experimental resin composites

Resins were made following the compositions given in Table 1. The used monomers were weighed and mixed with magnetic stirring. Experimental resin composites were made by mixing 70 wt% silica fillers (Series A: Schott & Series B: Esschem) with each resin matrix (30 wt%) using a high-speed mixing machine (SpeedMixer, DAC150 FVZ-K, Hauschild, Germany) with a speed of 1800 rpm. RI of the resins and polymers were calculated according to previous research [14].

**Table 1.** Classification of the experimental resins used in the study according to their composition and calculated refractive index (RI).

Experimental groups	Bis-GMA (wt%)	TEGDMA (wt%)	Camphorquinone (wt%)	DMAEMA (wt%)	RI
G1	20	80	0.7	0.7	1.477
G2	30	70	0.7	0.7	1.485
G3	40	60	0.7	0.7	1.493
G4	50	50	0.7	0.7	1.501
G5	60	40	0.7	0.7	1.508
G6	70	30	0.7	0.7	1.516
G7	80	20	0.7	0.7	1.524

### Translucency measurement (TP)

Specimens (*n* = 5) form each experimental resin composite (1 mm-thick rings with a diameter of 10 mm) were prepared. Curing of the resin composite was done using a hand light-curing unit (Elipar TM S10, 3 M ESPE, Germany) for 20 s in five separate overlapping portions from one side of the mold. The wavelength of the light was between 430 and 480 nm and light intensity was 1200 mW/cm^2^ (Marc Resin Calibrator, BlueLight Analytics Inc., Canada). After curing, the specimens were stored dry (24 h) at room temperature prior to testing.

Specimens color was assessed based on CIELAB color scale relative to the standard illuminant D65 over a black tile (CIE L* = 0, a* = 0.01 and b* = 0.03) and a white tile (CIE L* = 99.25, a* = −0.09 and b* = 0.05) on a reflection spectrophotometer (CM-700d, Konica-Minolta, Japan). The size of aperture was Ø 3 mm, and the illuminating and viewing configuration was CIE diffuse/10° geometry with the specular component included (SCI) geometry.

The translucency of the resin composites was obtained by calculating the color difference between the specimen over the white background and the specimen over the black background:

$$\mathrm{TP}={\left[{\left(L_{W^{*}}-L_{B^{*}}\right)}^{2}+{\left(a_{W^{*}}-a_{B^{*}}\right)}^{2}+{\left(b_{W^{*}}-b_{B^{*}}\right)}^{2}\right]}^{1/2}$$

where the subscript ‘W’ refers to the color coordinates over the white background and the subscript ‘B’ refers to those over the black background.

### Color matching evaluation (visual analysis)

**Figure 1.**
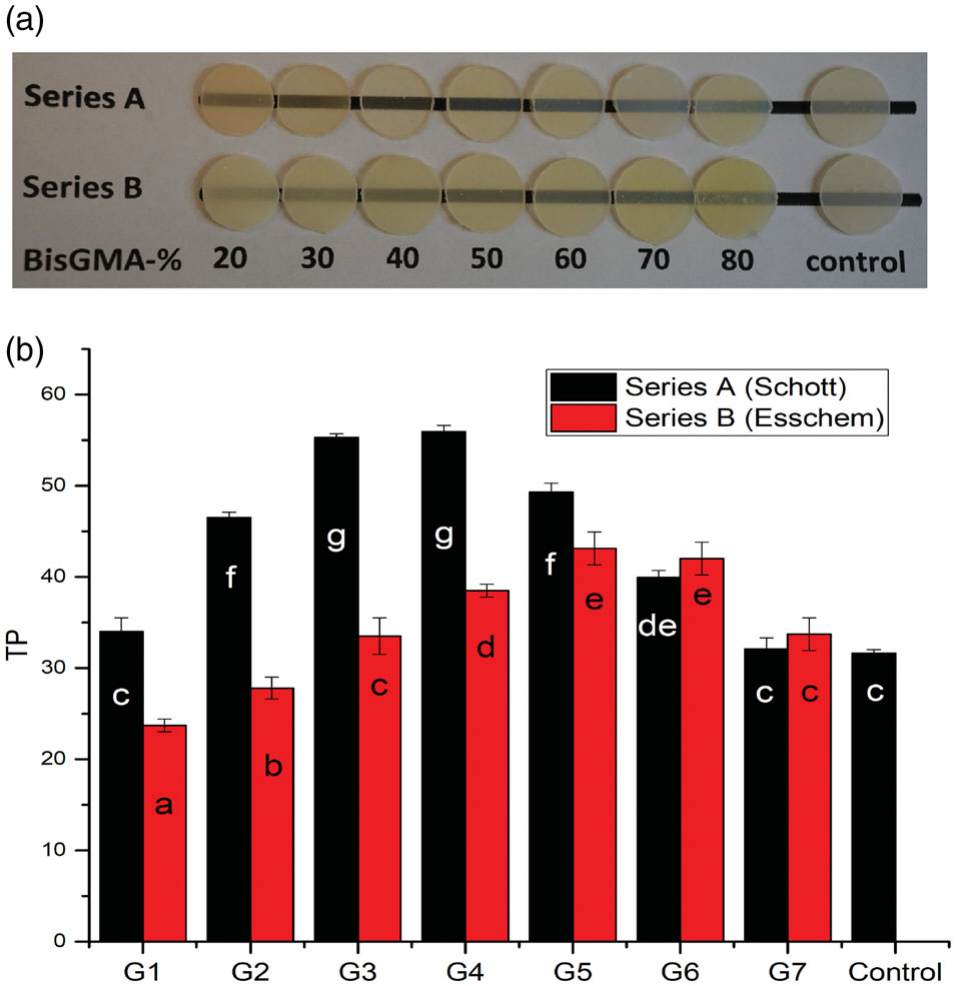
(a) Visual image of 1 mm thick resin composite specimens placed over a black line. (b) Translucency parameter mean values (TP) of experimental resin composites. Vertical lines represent standard deviation. The same letters inside the bars represent non-statistically significant differences (*p* > .05) among the materials.

Plastic crowns (*n* = 4/shade) simulating teeth were prepared from hybrid resin composite (DenFil, Vericom Corp., Korea) using three different shades (A1, A3, A4). Each crown had a round-shape cavity (Ø 4 mm) with 2 mm depth in the middle of the occlusal surface. Cavities were filled with control single-shade resin composite (Omnichroma), G1A, G7A and another commercial multi-shade resin composite with shade A3 (Filtek Supreme, 3 M, St Paul, MN). G1A and G7A were selected based on TP values which were closed to the control group (Figure 1). Due to the yellowish appearance or color, G3B and G7B were omitted from this test (Figure 1(a)).

Color matching was evaluated visually by three evaluators (LL, SG and MO). Under indoor light source with neutral light-gray walls and floor and using a 0°/45° viewing geometry, the evaluators performed blind visual evaluations (viewing distance ≈ 30 cm) of all restorations in a random order. The color differences between each crown and restoration were graded from 0 to 4, using the scale based on previous studies [5,15] where level ‘0’ means excellent match; 1, very good match; 2, not so good match (border zone mismatch); 3, obvious mismatch and 4, huge (obvious) mismatch.

### Statistical analysis

The results were examined by analysis of variance (ANOVA) with SPSS version 23 (SPSS, IBM Corp.) at the *p <* .05 significance level accompanied by a Tukey HSD *post hoc* test to determine the differences between the groups. Cronbach's alpha test was used to identify the reliability of color match between evaluators.

## Results

The RI of the experimental resin composites is summarized in Table 2. The results of translucency are presented in Figure 1(b). Data showed that composition of resin and type of fillers had a statistically significant effect on TP values (*p* < .05). In series A, the highest TP values were achieved around of 50–50% fractions of Bis-GMA and TEGDMA, while in series B were around of 60–40% fraction of Bis-GMA and TEGDMA. Comparing the TP values between all experimental resin composites, only G1B and G2B resin composite showed statistically lower TP values than control (*p* < .05). However, these differences can hardly be recognized visually (Figure 1(a)). Furthermore, the highest TP was encountered when the mismatch between the RI of fillers and resin (G4A: 1.53–1.501 = 0.03; G6B: 1.54–1.508 = 0.032) is around 0.03.

**Table 2.** Calculated refractive indices of the experimental resin composites.

Experimental groups	Series A	Series B
G1	1.514	1.521
G2	1.517	1.524
G3	1.519	1.526
G4	1.521	1.528
G5	1.523	1.530
G6	1.526	1.533
G7	1.528	1.535

Series A: 30 wt% resin + 70 wt% Schott fillers (RI 1.53).

Series B: 30 wt% resin + 70 wt% Esschem fillers (RI 1.54).

**Figure 2.**
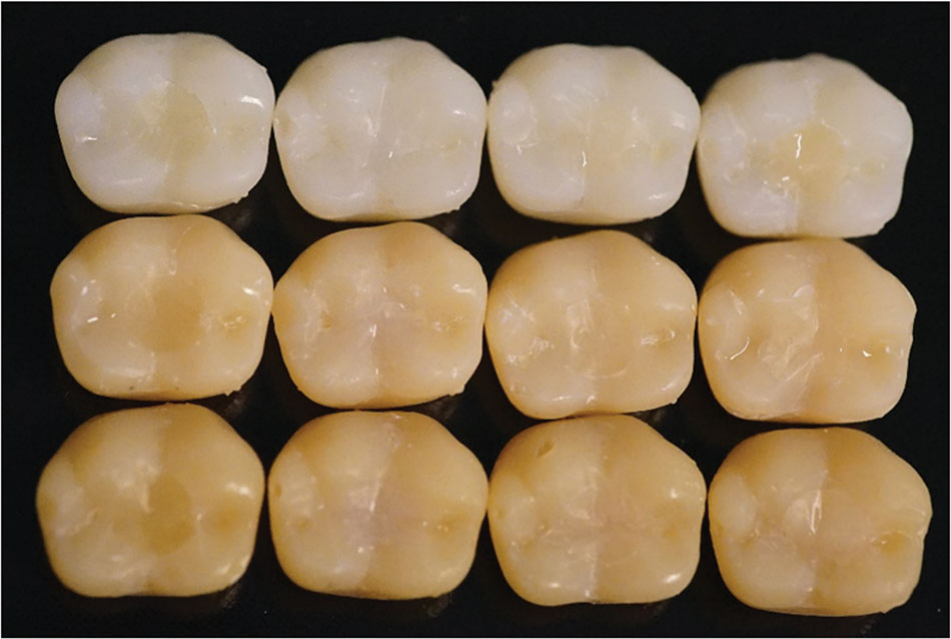
Representative images of different colored plastic crowns with composite restorations for visual color matching analysis. From left to right: G1A, G7A, Omnichroma, Filtek Supreme (shade A3). Shades from up to down: A1, A3, A4.

For visual evaluation of color matching (Figure 2), the Cronbach's alpha for the reliability test between evaluators was 0.821. Experimental G7A resin composite was behaving similarly to Omnichroma resin composite (single-shade, control), showing good color match with surrounding structure (chameleon effect). While G1A and Filtek Supreme (multi-shade) resin composites had clear color discrepancy with surrounding structure (Figure 2).

## Discussion

To obtain a high translucency of dental resin composite, matching the refraction index (RI) of the filler particles and the resin matrix has an important role. Hence, modifying the RI of the matrix to fit the filler particles was studied. In the present investigation, quite translucent experimental resin composites were developed. The assessment and explanation of the experimental results rejected the null hypotheses. It can therefore be claimed that the RI of the fillers and resin system affected the light scattering and translucency of dental resin composite and this would affect the composite color-matching or blending with surrounding tissues. Our results are in accordance with the findings of Ota et al., who reported a significance correlation between TP values and RI of resin composite [11]. The translucency enhanced and the opacity decreased as the RI variation between the resin and the filler decreased. However, in addition to the filler refractive index, the optical properties are influenced by filler loading, silane coupling of fillers and morphology, including filler particle size and distribution [16]. Though, in the absence of fillers, the monomers optical properties alter during polymerization as the RI rise that accompanies polymerization and light scattering is associated with vitrification and gelation [14,17].

In this study, the translucency of resin composites was increased with increasing the Bis-GMA fraction up to 50–60 wt%, and then was gradually decreased (Figure 1(b)). A linear relationship between the percentage of Bis-GMA in the resin matrix and the translucency of the material was stated earlier [18]. Due to the fact that Bis-GMA (RI = 1.54) has a refractive index similar to that of the silica filler (RI = 1.53) than that of TEGDMA (RI = 1.46), the difference in translucency may be attributable to this.

The chameleon or blending effect in dentistry concern to the interaction of restorative materials and surrounding hard dental tissues and is presented by a little color difference if they are seen together than if seen separately [19]. It means, two colors, viewed side by side, will blend under the suitable conditions: the perceived color of a region changes towards the color of the surrounding area. For the clinician, the chameleon effect deeds because it eliminates, minimizes, or neutralizes color mismatches and/or the lack of sufficient shade in the restorative material.

This pilot study was planned to determine the color change of resin composite restorations after placement in cavities in plastic teeth, since collecting many extracted teeth having similar color was not possible. The findings achieved from the visual color match assessment (Figure 2) seem to agree with the previous study, where single-shade resin composite (Omnichroma) exhibited the most pronounced color blends with surrounded structures [5]. On the other hand, this is in opposition to another study, where omnichroma displayed inferior color-matching ability to a multi-shade resin composite [20]. Interestingly, our experimental G7A resin composite having calculated RI of 1.52 was behaving similar than omnichroma resin composite (Figure 2). According to manufacturer, omnichroma has RI of 1.47 and 1.52 before and after curing, which means both materials have similar light scattering and absorption. Although, omnichroma has no Bis-GMA in its resin matrix and composed of 260 nm (79 wt%) spherical silicon/zirconium dioxide fillers [20]. This finding was confirmed by the measured TP values (G7A and control) (Figure 1) and in line with the previous investigation that found a strong correlation between the blending effect related to color shifting and the TP values of resin composite [8].

Various colors are viewed since objects reflect some light and absorb others. Reflection occurs when light beams hit an object and are reflected from the surface (surface reflection) or (for high translucent objects) deeper layers [5]. It is important to note that enamel has RI of 1.63 which is higher than most of the existence resin composites [21]. Therefore, in order to have good color blending or matching at the enamel borders of composite restorations, certain translucency and RI of resin composite are needed.

Visual color determination of a patient’s tooth is the most frequently applied method in clinical dentistry [5]. However, visual color assessment has been found to be unreliable and subjective that people try to overcome [22,23]. Visual color assessment is dependent on the observer’s physiologic and psychologic responses to radiant energy stimulation [20]. Inconsistencies may result from uncontrolled factors such as fatigue, aging, emotions, lighting conditions, previous eye exposure, object and illuminant position and metamerism [23]. On the other hand, instrumental color analysis offers a potential advantage over visual color determination because instrumental readings are objective, can be quantified and are more rapidly obtained. As stated by Della Bona et al., shade training and/or dental experience are an important component in color matching agreement between visual–instrumental identifications [23].

Several factors affect the way the color match is viewed in patient’s mouth, including the area where the tooth is restored, the morphology of the tooth, the influence of the soft tissues and saliva surrounding it, among others. Furthermore, natural teeth are translucent, multilayered, and curved, which influences the manner of light reflecting or scattering.

Within the limitations of this study, including fillers having RI of 1.53 into BisGMA/TEGDMA resin with refraction index of 1.524 resulted in composite resin providing good color match with surrounding structure ‘chameleon effect’.
